# Adenovirus Precursor pVII Protein Stability Is Regulated By Its Propeptide Sequence

**DOI:** 10.1371/journal.pone.0080617

**Published:** 2013-11-15

**Authors:** Raviteja Inturi, Srinivas Thaduri, Tanel Punga

**Affiliations:** Department of Medical Biochemistry and Microbiology, Uppsala University, Uppsala, Sweden; University of Regensburg, Germany

## Abstract

Adenovirus encodes for the pVII protein, which interacts and modulates virus DNA structure in the infected cells. The pVII protein is synthesized as the precursor protein and undergoes proteolytic processing by viral proteinase Avp, leading to release of a propeptide sequence and accumulation of the mature VII protein. Here we elucidate the molecular functions of the propeptide sequence present in the precursor pVII protein. The results show that the propeptide is the destabilizing element targeting the precursor pVII protein for proteasomal degradation. Our data further indicate that the propeptide sequence and the lysine residues K26 and K27 regulate the precursor pVII protein stability in a co-dependent manner. We also provide evidence that the Cullin-3 E3 ubiquitin ligase complex alters the precursor pVII protein stability by association with the propeptide sequence. In addition, we show that inactivation of the Cullin-3 protein activity reduces adenovirus E1A gene expression during early phase of virus infection. Collectively, our results indicate a novel function of the adenovirus propeptide sequence and involvement of Cullin-3 in adenovirus gene expression.

## Introduction

Adenoviruses (Ad) are non-enveloped, double-stranded DNA viruses that infect a wide range of species. The virus particle is composed of an outer capsid shell, where the fiber and penton proteins are needed for the attachment to the recipient cell receptors. Internalization of the virus particle occurs via clathrin-mediated endocytosis, followed by release of partially uncoated capsid into the cytoplasm [1]. In addition to providing the interaction surface, the capsid is also essential for protecting the internal viral core structure of the particle. Inside the core, viral DNA is associated with the viral core proteins V, VII, Mu, IVa2 and the terminal protein. In addition, the viral DNA-dependent adenovirus proteinase (Avp), has been considered as an essential component of the viral core [2]. Among these proteins, the protein VII is the major core protein, showing a very stable association with the viral DNA [3-5]. 

In human adenoviruses, protein VII is encoded as a precursor protein, designated as the pVII protein [6]. Similarly to some other adenoviral proteins (IIIa, pVI, pVIII, pTP and polypeptide X), pVII undergoes a site-specific proteolytic cleavage by the Avp during the final steps of virus maturation. The site-specific cleavage of the precursor pVII protein results in release of a short N-terminal propeptide (amino acids 1-24 in the case of Ad2/Ad5) and in accumulation of the mature VII protein [7,8]. The mature VII protein is rich in basic amino acids and this particular feature has been implicated in its high affinity interaction with the viral DNA [4,9,10]. Similarly to cellular histones, mature VII wraps viral DNA into nucleosome-like structures, introduces superhelical turns into DNA and condenses DNA, indicating that it can function as a viral histone-like protein [5,11]. The mature VII protein can serve several functions for the virus. First, the mature VII protein has been suggested to function as the adaptor molecule needed for viral DNA import into the nucleus at the onset of infection [12]. Second, mature VII can protect the incoming viral DNA from cellular DNA damage response activated during early times of infection [13]. Third, mature VII controls expression of the viral genes. The incoming viral DNA is associated with the mature VII protein, which has been indicated to function as the transcription silencing factor [14]. To overcome the potential negative effect of VII, the viral chromatin structure has to be remodelled to allow efficient viral gene expression and DNA replication. The remodelling steps can involve gradual loss of mature VII from virus chromatin [15,16] or the recruitment of cellular template activating factor TAF-1β/SET to viral DNA [17-19].

Protein ubiquitination is one of the best-studied post-translational modifications in the cell. The covalent attachment of the ubiquitin moiety can target substrate proteins for proteolytic degradation via ubiquitin-proteasome system (UPS) [20]. Ubiquitin attachment is catalyzed by sequential involvement of ubiquitin-activating enzyme (E1), ubiquitin-conjugating enzyme (E2) and ubiquitin ligase (E3). Importantly, E3 confers the specificity to ubiquitination by mediating the interaction between the substrate protein and an E2 [21]. Among the characterized E3 ligases, the Cullin-RING E3 ubiquitin-Ligases (CRL) have been considered as the most prominent class of multisubunit E3 ubiquitin-ligases [22]. The key proteins in CRL are the Cullins (Cul1-7 in mammals), which function as the molecular scaffolds to position the substrate proteins in close proximity to the E2 enzymes, which in turn couple the ubiquitin to the substrate protein. Several viruses target UPS for their efficient replication in the host cells [23]. In the case of human adenoviruses the best-known example is the Ad5 E1B55K/E4orf6 protein complex, which by recruiting Cul5-based CRL complex to the cellular p53, Mre11, DNA ligase IV, integrin α3, Tip60 and ATRX proteins promotes their proteolytic degradation [24-29]. The viral proteins can also be the substrates for ubiquitination. For instance, the adenovirus protein VI binds and is ubiquitinated by the Nedd4 E3 ubiquitin ligase, which facilitates adenovirus particle trafficking in the cytoplasm [30]. In addition, the protein VI association with Nedd4 has been shown to promote initial expression of the early Ad5 E1A gene by counteracting the cellular transcriptional repressor protein Daxx [31].

Here we show that the Ad5 pVII protein stability is regulated by the proteasomal pathway. We show that the propeptide sequence present in the precursor pVII protein is the destabilizing element of the protein. The propeptide sequence showed an enhanced interaction with the Cul3 protein and inhibition of the Cul3 enzymatic activity resulted in an increased stability of the precursor pVII protein. We have also identified two lysine residues, K26 and K27, which can control the Ad5 precursor pVII protein stability. In addition, we demonstrate the involvement of the functional Cul3 protein in adenovirus E1A gene expression during early phase of the infection. 

## Materials and Methods

### Cell culture and viruses

HEK293T and HeLa cells [32] were cultured in Dulbecco’s Modified Eagle Medium (DMEM, Invitrogen) supplemented with 10% FCS (PAA) and penicillin-streptomycin solution (Gibco). The stable HEK293 cell lines (HEK293-pVII(wt)Flag and HEK293-pVII(Δ24)Flag), expressing codon-optimized pVII(wt)Flag and pVII(Δ24)Flag proteins, were generated by using Flp-In™ T-Rex™ system (Invitrogen). The stable HeLa cell line expressing DN-Cul3-Flag protein was generated by using HeLa-Flp-In™ T-Rex™ cell line, generously provided by Prof. Stephen Taylor, Manchester, United Kingdom [33]. The expression of the proteins in stable cell lines was induced with doxycycline (Sigma, D9891) at final concentrations of 0.2 μg/ml. All cell lines were grown in a humidified chamber with 7% CO_2_ at 37°C. The human Ad5(wt) virus used in the study was obtained from Prof. Göran Akusjärvi, Uppsala University, Sweden. Cells were infected with virus at a multiplicity of infection (MOI) of 5 fluorescence-forming units (FFU) per cell. 

### Plasmid constructions and transfections

The original, Ad5 derived pVII(wt) cDNA was obtained from Dr. Harry Wodrich, Montpellier, France. To enhance the expression of pVII(wt) in mammalian cells, the mRNA sequence was codon-optimized with OptimumGene™ Codon Optimization Technology (GenScript USA Inc.). To detect the expression of codon-optimized precursor pVII as the C-terminal Flag-tag fusion protein, the cDNA was cloned into pcDNA3.1(+)Flag vector [32]. To obtain mature VII (pVII(Δ24)) expression vector, codon optimized pVII cDNA was amplified with forward primer 5´-agtgaattcgccacc**atg** gcaaaaaagaggtcagac-3´and reverse primer 5´-gccgtcgacatttctagggggcctg-3` and cloned into EcoRI/XhoI sites in pcDNA3.1(+)Flag vector. Nucleotides marked in bold represent the first methionine codon, followed by codon-optimized pVII sequence starting at the 25^th^ codon (Ala) (underlined). Point mutations into the codon-optimized precursor pVII and mature VII sequence were generated by using QuickChange Lightning Site-Directed Mutagenesis Kit (Agilent Technologies). The plasmid expressing 1-24Gal4 fusion protein was created by inserting codon-optimized propeptide cDNA sequence (1-24 amino acids of Ad5) in-frame with the 5´end of the Gal4 DNA binding domain sequence [34]. The generated 1-24Gal4 fusion sequence was cloned into pcDNA3.1(-) vector (Invitrogen). To express the GST-fusion proteins, codon-optimized pVII(wt), pVII(K26R/K27R) and pVII(Δ24) sequences were cloned into pGEX-6P-1 (GE Healthcare Life Sciences) vector. All pVII sequence-containing plasmids were sequenced to verify the correctness of the inserts. Plasmids expressing DN-Cul1-5-Flag proteins have been described previously [32] and were originally kindly provided by Dr. Wade Harper [35]. Firefly and Renilla luciferase reporter gene plasmids, pTK-MH100x4-Luc and pCiNeoControl, were generously provided by Drs. Ronald Evans and Witold Filipowicz, respectively. The plasmid encoding for the Flag-Cul3(wt) protein was received from Prof. Chin Ha Chung, Seoul, Korea [36]. Transient transfections were performed with the jetPRIME (Polyplus Transfection) reagent in HEK293T cells and with the Turbofect reagent (Thermo Scientific) in HeLa cells. Most of the transfections were performed on 24-well plates with approximately 60-80% cell confluence on the day of transfection. RNAi experiments were conducted by transfecting ON-TARGETplus SMART pool siRNAs (Thermo Scientific) against Cul3, Psmb1 and non-target control (Scr) at final concentration of 20 nM for 36 hours with the Dharmafect 1 reagent (Thermo Scientific). Thereafter expression of the pVII(wt)-Flag protein was induced with doxycycline for additional 14 hours. 

### RNA extraction and qRT-PCR

Total RNA extraction and cDNA synthesis was performed as published previously [37]. Purified RNA was also treated with DNaseI (RapidOut DNA Removal Kit; Thermo Scientific) to remove genomic DNA from the RNA preparations before performing cDNA synthesis. RNA expression was analysed by quantitative reverse transcription PCR (qRT-PCR) on an Applied Biosystems 7900 system (Life Technologies) using HOT FIREPol^®^ EvaGreen^®^ qPCR Supermix (Solis BioDyne). Primer sequences are available in Supporting Information (Table S1).

### Antibodies and chemicals

The following antibodies were used for Western blotting throughout the study: anti-mouse Flag (Sigma, M2, F1804), anti-rabbit Flag (Sigma, F7425), anti-rabbit Gal4 (Abcam, ab1396), anti-goat Actin (Santa Cruz, sc-1616), anti-rabbit Cullin-3 (Sigma, C9745), anti-rabbit GST (Santa Cruz, sc-33614), anti-mouse E1A (EMD Millipore, DP11-UG100), anti-c-Myc (Santa Cruz, sc-42) and anti-Ad5 capsid (Abcam, ab6982). To inhibit proteasome MG132 (Sigma, C2211, dissolved in DMSO) was used at final concentration of 25 µM for 3-5 hours or at final concentration of 5 µM for 12 hours. Control cells for MG132 treatment were treated only with DMSO. Protein stability experiments were performed by using cycloheximide (Sigma, C4859, dissolved in DMSO) at final concentration of 100 µg/ml. 

### GST-pull-down assay

Plasmids expressing the GST-pVII(wt), GST-pVII(K26R/K27R), GST-pVII(Δ24) and GST proteins were transformed into BL21-CodonPlus (DE3)-RIPL cells (Agilent Technologies). The expression of the proteins was induced with 0.1 mM IPTG for 16 hours at 25°C. The GST-tagged proteins were purified according to the published protocol [12]. To obtain whole cell lysate containing Flag-Cul3(wt) protein, HEK293T cells on 100 mm cell culture plates were transfected with plasmid expressing Flag-Cul3(wt) for 48 hours. Cells were collected and lysed in buffer A (25 mM Hepes-KOH (pH 7.4), 12.5 mM MgCl_2_, 100 mM KCl, 0.1 mM EDTA, 10% glycerol, 0.1% NP-40, supplemented with protease inhibitor (Complete Mini EDTA-free, Roche Applied Sciences)) for 30 minutes on ice. Obtained soluble cell lysates were incubated with 2 μg of GST fusion proteins at room temperature for 1 hour. The beads were washed extensively with buffer B (25 mM Hepes-KOH (pH 7.4), 12.5 mM MgCl_2_, 200 mM KCl, 0.1 mM EDTA, 10% glycerol, 0.1% NP-40) and the bound proteins were separated on 12% SDS-PAGE, followed by detection of the proteins by Western blotting.

### Peptide-pull-down assay

Control (Biotin-CPPSAELYSNALPV) and 1-24 propeptide (Biotin-MSILISPSNNTGWGLRFPSKMFGG) peptides were purchased from GenScript USA Inc. Five micrograms of both peptides were coupled to Dynabeads MyOne Streptavidin T1 magnetic beads (Invitrogen) and incubated with HEK293T whole cell lysate expressing the Flag-Cul3(wt) protein. The binding reactions were done in NET buffer (50 mM Tris-HCl (pH 8.0), 150 mM NaCl, 0.5 % TritonX-100) at room temperature for 30 minutes on end-over-end rotator. The magnetic beads were washed with 4x1ml in NET buffer and the bound proteins were resolved on 10% SDS-PAGE.

### Luciferase assay

HEK293T cells were cotransfected with 50 ng of reporter plasmids pTK-MH100x4-Luc and pCiNeoControl in the presence of increasing amounts (150-300 ng) of 1-24Gal4 encoding plasmid or with Gal4 plasmid (300 ng). Cells were lysed with Passive Lysis Buffer (Promega) and the expression of target Firefly luciferase (pTK-MH100x4-Luc) and control Renilla luciferase (pCiNeoControl) proteins were detected by using Dual-Luciferase® Reporter Assay System (Promega). The target Firefly luciferase values were normalized to the control Renilla luciferase values and the data from three independent experiments performed in duplicates is shown.

### Western blot analysis

The cell lysates were prepared by disrupting the cell pellets in RIPA buffer (25 mM Tris-HCl (pH 7.4), 150 mM NaCl, 1% NP-40, 1% sodium deoxycholate, 0.1 % SDS, supplemented with protease inhibitor (Complete Mini EDTA-free, Roche Applied Sciences)) after 36 hours of transfection. After incubating the lysates on ice for 30 min, the samples were sonicated with Bioruptor^®^ (Diagenode) and centrifuged at 13000 rpm for 10 minutes at 4°C. Protein concentration was measured with Bradford Assay (BioRad) and equal concentration of proteins were separated on SDS-PAGE. The proteins were transferred onto nitrocellulose membrane (Protan) by using wet-transfer system (BioRad). The membrane was blocked in Odyssey blocking buffer (LI-COR) and thereafter incubated with the primary antibody diluted in the blocking buffer overnight at 4°C. After intensive washing with 1X PBS-T (PBS+0.1% Tween 20), the membranes were incubated with the fluorescent secondary antibodies (IRDye®, LI-COR) for 1 hour at room temperature. The membranes were scanned and the fluorescence signals were detected by using the Odyssey scanner (LI-COR). The protein quantification was done by using Image Studio Software (LI-COR).

### Indirect immunofluorescence assay

Indirect immunofluorescence assays were carried out as described previously [38]. Briefly, HeLa cells were grown on fibronectin coated coverslips and transfected for 24 hours with the indicated plasmids. Cells were fixed with 3% paraformaldehyde in PBS for 15 minutes at room temperature and permeabilized with 0.1% Triton X-100 in PBS-T (PBS+0.01% Tween 20) for 15 minutes at room temperature. After blocking the cells with 2% BSA/PBS-T solution, the coverslips were subjected to immunofluorescence analysis using anti-Flag (M2, Sigma, 1:1000) and anti-fibrillarin (Abcam, ab5821, 1:500) antibodies. Proteins were visualized with anti-FITC-conjugated anti-rabbit IgG (Sigma, F6005, 1:1000) and anti-TRITC-conjugated anti-mouse IgG (Sigma, T5393, 1:1000) secondary antibodies. Nucleus was detected by DAPI (1μg/ml) staining supplemented into the Floromount-G mounting media (Southern Biotech). Labelled cells were visualized by a fluorescence microscope (Nikon eclipse 90i) and the images were analysed with NIS-elements (AR 3.10, Nikon) software. 

## Results

### Adenovirus pVII propeptide sequence induces proteasomal degradation

**Figure 1:**
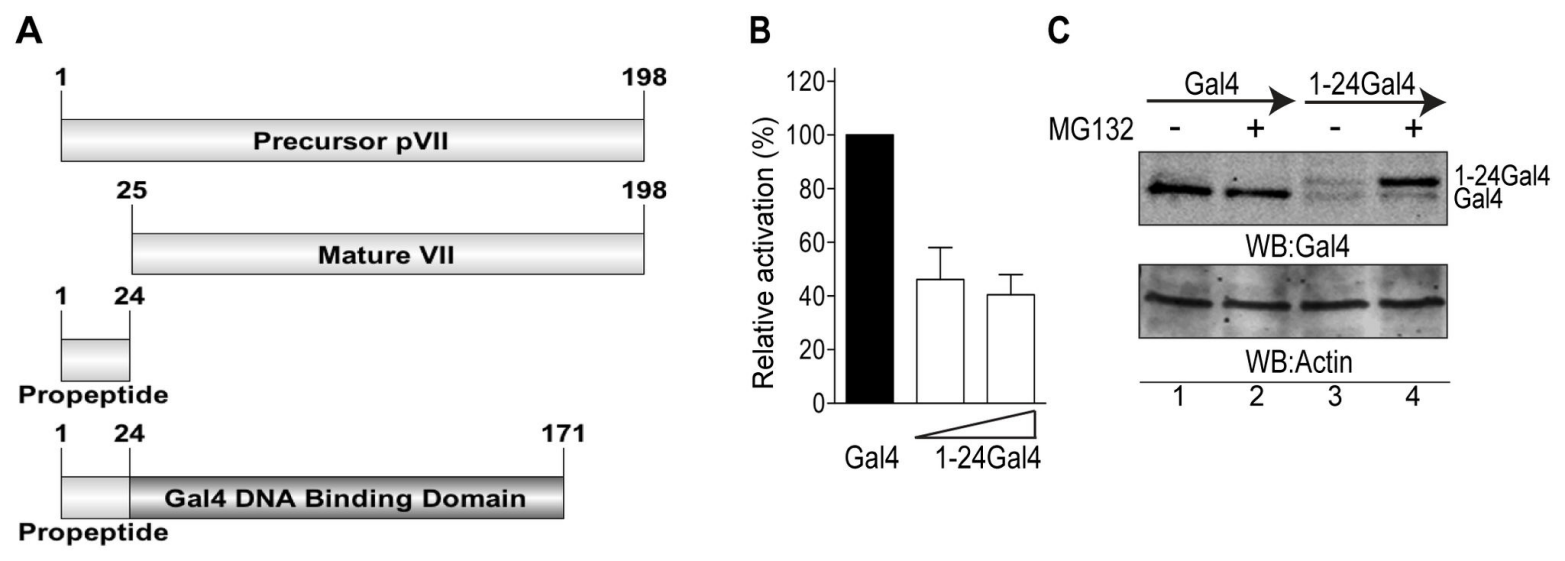
Adenovirus pVII propeptide sequence induces proteasomal degradation. (**A**) Schematic representation of the Ad5 precursor pVII, mature VII, propeptide and 1-24Gal4 proteins. (**B**) Repression of Gal4 DNA binding site containing luciferase reporter by the 1-24Gal4 protein in HEK293T cells. Data shown as relative activation of Firefly luciferase reporter gene after normalization to Renilla luciferase control gene. (**C**) MG132 treatment increases the 1-24Gal4 protein levels. HEK293T cells expressing the Gal4 and 1-24Gal4 proteins were treated with 25 μM of MG132 for 5 hours. Protein expression was detected by Western blotting (WB) using anti-Gal4 and anti-Actin antibodies. Presence of the Gal4 protein signal in 1-24Gal4 transfected cells is due to internal translation initiation of the Gal4 protein encoded by the 1-24Gal4 plasmid.

Several adenoviral proteins undergo site-specific proteolytic cleavage by the viral proteinase Avp during the late stage of infection [2]. Avp cleavage of pVII results in a release of a short N-terminal propeptide and accumulation of the mature VII protein (Figure 1A). Whereas the mature VII protein has established functions on viral DNA, the importance of the propeptide in the context of the precursor pVII protein remains unclear. To gain insight into a potential function of the propeptide we decided to test the ability of the propeptide to regulate reporter gene expression, due to its previously described DNA-binding activity [4]. An expression vector was generated where the propeptide sequence (amino acids 1-24 of Ad5) was fused to the N-terminus of Gal4 DNA binding domain generating the 1-24Gal4 fusion protein. This protein mimics size-wise the precursor pVII protein and at the same time can be specifically recruited to DNA via the Gal4 DNA-binding motif (Figure 1A). The activity of the 1-24Gal4 fusion protein was analysed on Gal4 binding site containing reporter plasmid expressed in HEK293T cells. The results showed a reduction of the reporter gene activity in presence of the 1-24Gal4 fusion protein (Figure 1B). Following Western blot analysis indicated that the 1-24Gal4 protein was expressed at much lower levels compared to the Gal4 protein (Figure 1C, lanes 1 and 3), suggesting that reduced reporter gene activity was due to diminished fusion protein levels. To test if addition of the propeptide to Gal4 creates an unstable fusion protein, we treated the 1-24Gal4 protein expressing HEK293T cells with the proteasome inhibitor MG132 and analysed protein accumulation by Western blotting. Surprisingly, the 1-24Gal4 protein was clearly detected after MG132 treatment, whereas the Gal4 protein levels were not enhanced by the same treatment (Figure 1C). In summary, our data indicated that the propeptide derived from the Ad5 pVII precursor protein can function as the protein stability regulating sequence.

### Propeptide sequence controls the Ad5 precursor pVII protein stability

**Figure 2:**
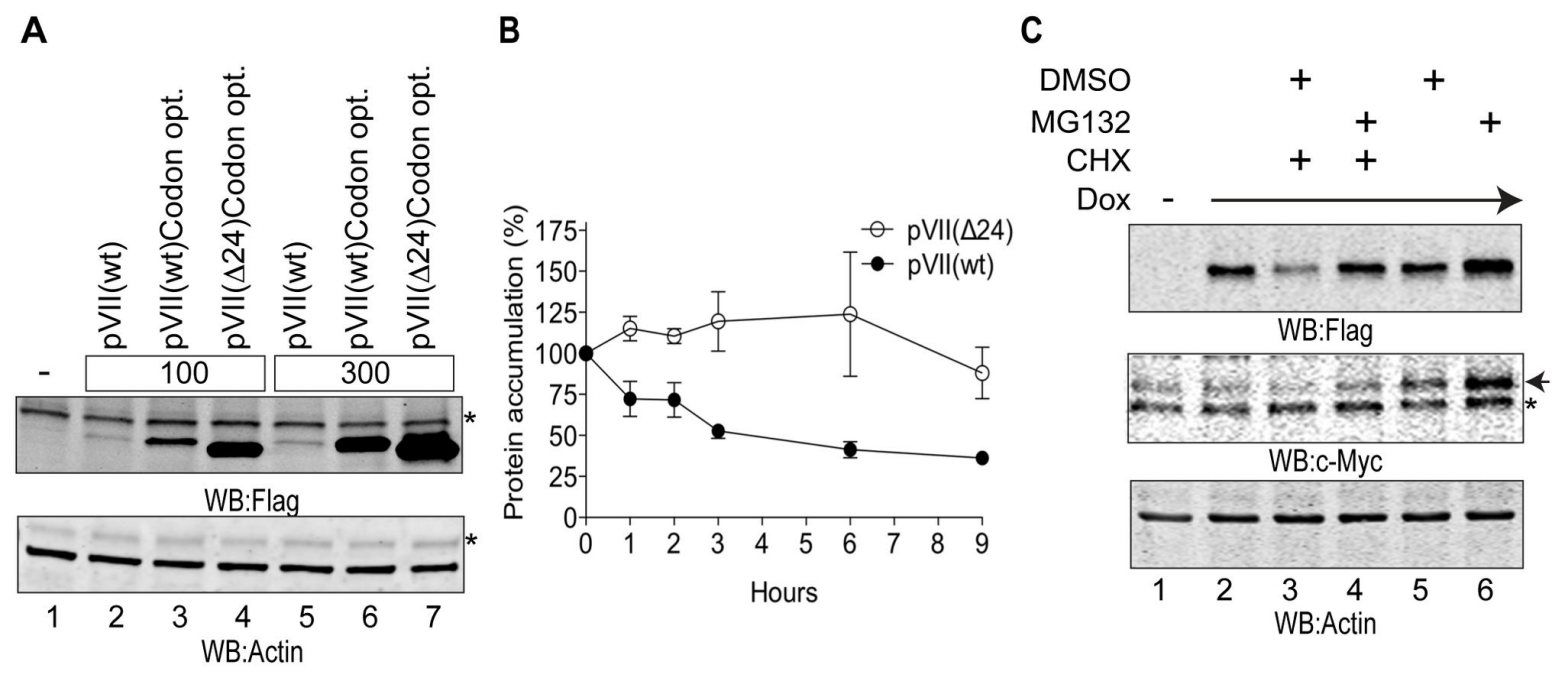
Propeptide sequence controls the Ad5 precursor pVII protein stability. (**A**) Codon optimization of pVII sequence enhances the protein detection. HEK293T cells were transiently transfected with two different concentrations, 100ng (100) and 300ng (300), of the original Ad5 pVII(wt)Flag, codon-optimized (Codon opt.) Ad5 pVII(wt)Flag and Ad5 pVII(Δ24)Flag expressing plasmids. Protein expression was detected by Western blotting using anti-Flag and anti-Actin antibodies. (**B**) Decay of the pVII(wt)Flag protein is faster compared to the pVII(Δ24) protein. Doxcycline (Dox)-inducible HEK293 stable cell lines, expressing the pVII(wt)Flag and pVII(Δ24)Flag proteins for 12 hours, were treated with cycloheximide (CHX) (100µg/ml) for defined time points (1, 2, 3, 6 and 9 hours). Protein accumulation was quantitated by Western blotting and the Flag-tagged protein levels were normalized to Actin protein levels. Average from three independent experiments is shown. (**C**) Inhibition of proteasome enhances pVII(wt) detection. HEK293 stable cell line, expressing the pVII(wt)Flag protein after 12 hours of induction with Dox was treated with CHX (100µg/ml) for 3 hours (lanes 3 and 4). To block proteasomal activity the cells were simultaneously treated with MG132 (25μM) for 3 hours (lane 4). Protein expression was visualized by Western blotting using anti-Flag, anti-c-Myc and anti-Actin antibodies. Asterisk (*) indicates the detection of the unspecific proteins by the antibodies. Black arrow points to the migration of the c-Myc protein. Hyphen (-) indicates non-transfected or non-Dox induced cells.

 In adenovirus infected cells the propeptide is incorporated into the precursor pVII protein sequence. Thus, it was important to evaluate if the presence of the propeptide would also affect the precursor pVII protein stability. For this reason we decided to compare the expression levels of the precursor pVII (hereafter referred to as pVII(wt)) and the mature VII (hereafter referred to as pVII(Δ24)) proteins in mammalian cells. Despite several efforts to express Ad5 derived pVII cDNA in different cell lines, we were unable to detect high expression levels of the protein by Western blotting (Figure 2A, lanes 2 and 5). One reason for this failure could be a non-optimal codon bias of pVII mRNA for transient expression in mammalian cells. To circumvent this problem, we codon-optimized the pVII mRNA sequence, by exchanging approximately 74% of the sequence, meanwhile keeping intact the original amino acid sequence of the protein. Indeed, codon optimization significantly enhanced the detection of the pVII(wt) protein (Figure 2A, compare lanes 2 and 5 to lanes 3 and 6). Therefore, all the subsequent experiments were conducted with the plasmids expressing the codon-optimized Ad5 pVII sequence. Interestingly, the codon-optimized pVII(wt) and pVII(Δ24) proteins showed different accumulation patterns, where the pVII(Δ24) protein accumulated at higher levels compared to the pVII(wt) protein (Figure 2A, compare lane 3 to lane 4 and lane 6 to lane 7). To further analyse the protein stability we generated inducible HEK293 stable cell lines expressing the Flag-tagged pVII(wt) and pVII(Δ24) proteins in response to doxycycline treatment. In these cell lines the expression of both proteins was induced with doxycycline for 12 hours followed by treatment of the cells with cycloheximide. The latter is a protein synthesis inhibitor, which makes it possible to follow protein decay under experimental conditions where the *de novo* protein synthesis is inhibited. Interestingly, we observed a more rapid decay of the pVII(wt) protein compared to the pVII(Δ24) protein in this experimental system (Figure 2B and Figure S1). To understand if the faster decay of the pVII(wt) protein was due to enhanced proteasomal degradation, the pVII(wt) protein expressing cell line was treated with the proteasome inhibitor MG132. Truly, MG132 efficiently blocked the pVII(wt) protein degradation in cycloheximide-treated cells (Figure 2C, compare lanes 3 and 4). Similarly, a well-known unstable cellular protein c-Myc [39] responded to these treatments, confirming the proper function of the used chemicals (Figure 2C, middle panel). Taken together, these data pointed towards a potential involvement of proteasomal degradation regulating the pVII(wt) protein stability. 

### Lysines K26 and K27 control the Ad5 precursor pVII protein stability

**Figure 3:**
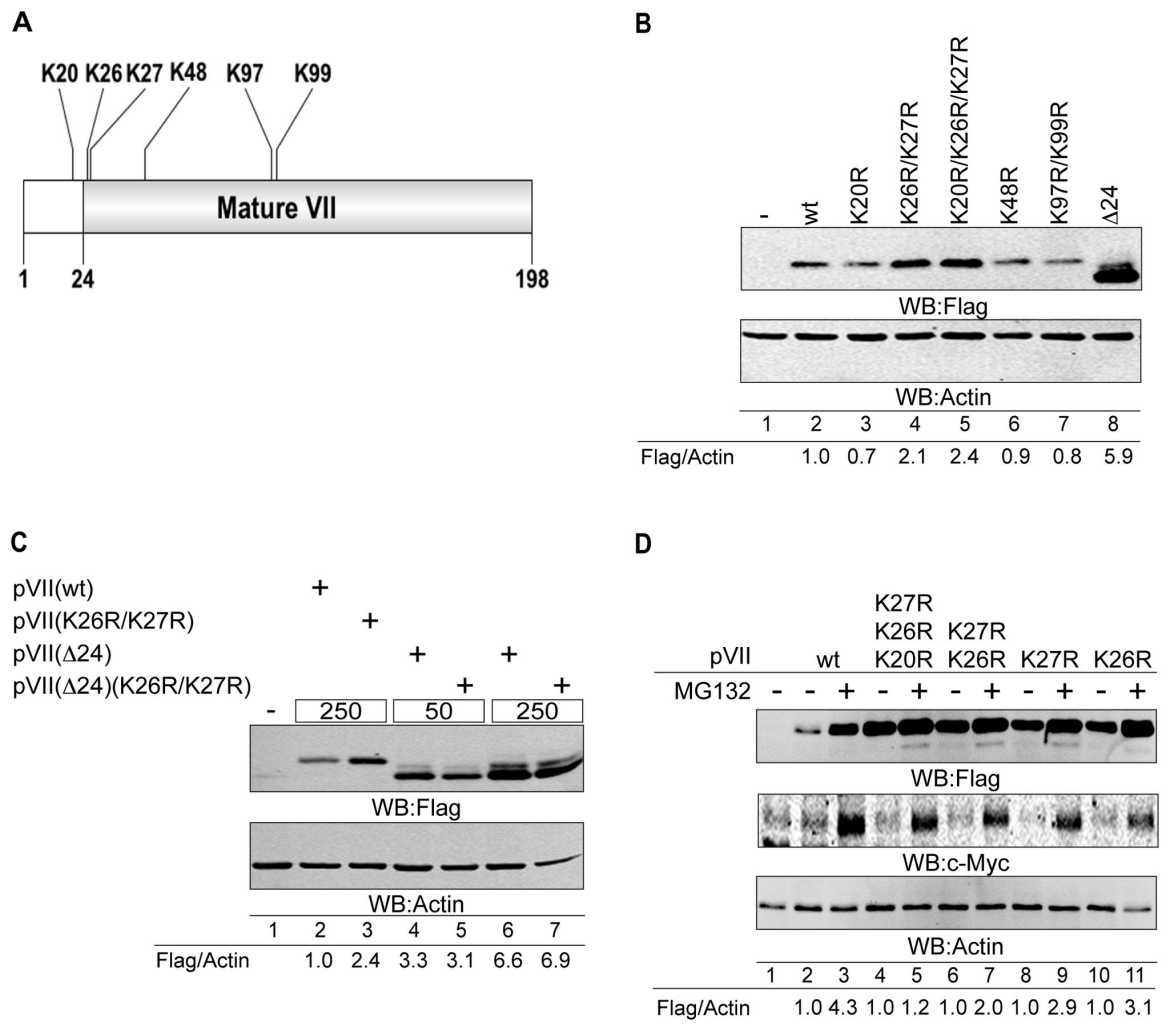
Lysines K26 and K27 control the Ad5 precursor pVII protein stability. (**A**) Schematic localization of Ad5 pVII lysine (K) residues in the protein. (**B**) Mutation of lysines K26 and K27 increase the pVII protein stability. The expression of Flag-tagged pVII lysine mutant proteins was detected in transiently transfected HeLa cells. The relative expression levels of the Flag-tagged pVII proteins are shown as the fluorescence signal ratio of Flag/Actin proteins. Hyphen (-) indicates non-transfected cells. (**C**) K26/K27 mutation in the pVII(Δ24) protein does not increase the protein stability. Indicated proteins were transiently expressed in HeLa cells and their accumulation was analysed by Western blotting. Two different plasmid concentrations, 50ng (50) and 250ng (250), were used to express the pVII(Δ24)Flag proteins. Quantification of the Flag-tagged pVII proteins is shown as the fluorescence signal ratio of Flag/Actin proteins. Hyphen (-) indicates non-transfected cells. (**D**) Accumulation of the pVII mutant proteins in the presence of MG132. HEK293T cells transiently transfected with the pVII mutant proteins expressing plasmids were treated with 5μM MG132 for 12 hours. The quantification of Flag-tagged pVII proteins, shown below the image, is mean of two independent experiments. MG132-treated signals were related to DMSO-treated signals (-), which were set as 1. In all samples the Flag signals were normalized to the Actin signal (Flag/Actin). Anti-c-Myc antibody was used to monitor the MG132 treatment.

Protein degradation via UPS is mainly based on ubiquitin attachment to lysine (K) residues on the substrate proteins. Ad5 pVII has in total six lysine residues in its protein sequence (Figure 3A). To analyse the importance of these lysine residues in the pVII(wt) protein stability, we generated plasmids where the amino acid codons encoding for lysines were exchanged to arginines (R). The generated plasmids were transfected into HeLa cells and the steady state pVII protein levels were monitored by Western blotting. Interestingly, mutations at K26 and K27 residues enhanced the accumulation of the pVII(wt) protein (Figure 3B). In contrast, mutating K20, K48, K97 and K99 residues did not notably elevate the pVII protein levels. Similarly to the experiment done in HEK293T cells (Figure 2A), the pVII(Δ24) protein levels increased also in HeLa cells (Figure 3B). Thus, the proteins where either the lysines K26 and K27 or the propeptide sequence were mutated or deleted showed enhanced accumulation. This observation urged us to study a potential co-dependent role of these sequence components in the pVII(wt) protein stability regulation. Therefore, we compared the protein accumulation pattern of the pVII(wt), pVII(K26R/K27R), pVII(Δ24) and pVII(Δ24)(K26R/K27R) proteins in transiently transfected HeLa cells. Interestingly, mutation of the K26 and K27 residues in the pVII(Δ24) protein background did not increased the protein stability (Figure 3C, lanes 4-7). This observation was in contrast with the accumulation pattern seen for the pVII(wt) and pVII(K26R/K27R) proteins (Figure 3C, lanes 2 and 3). To further validate the involvement of K26 and K27 in proteasomal degradation, we treated the HEK293T cells expressing different pVII lysine mutated proteins with the MG132 for 12 hours and followed their steady state levels by Western blotting. Similarly, to the previous experiment (Figure 2C) MG132 treatment increased the pVII(wt) protein levels in HEK293T cells (Figure 3D). In contrast, the proteins where both K26 and K27 were mutated the MG132 treatment did not show similar accumulation pattern as was seen with the pVII(wt) protein. However, the proteins containing single K26 and K27 mutations still responded to some extend to the MG132 treatment (Figure 3C). Taken together, the data indicated a potential co-dependent role of the propeptide sequence and the lysine K26, K27 residues in controling the stability of the Ad5 pVII(wt) protein.

### Cul3 E3 ubiquitin ligase reduces the Ad5 precursor pVII protein levels

**Figure 4:**
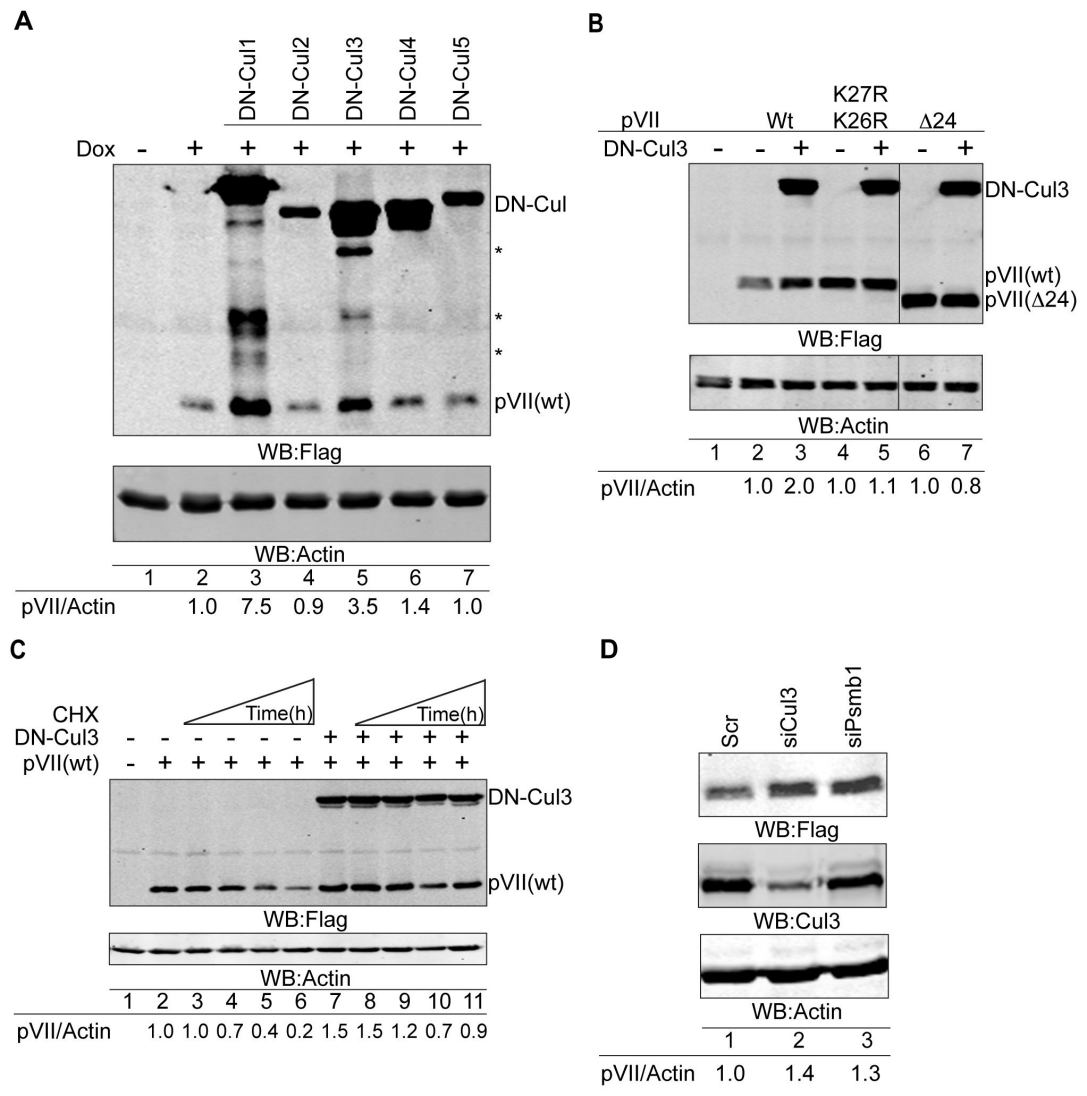
Cullin-3 E3 ubiquitin ligase reduces the Ad5 precursor pVII protein levels. (**A**) DN-Cul3 enhances the pVII(wt) protein stability. Expression of the pVII(wt)Flag protein was detected in Dox-induced HEK293-pVII(wt)Flag stable cell line in the presence of transiently overexpressed DN-Cul1-5 plasmids. The pVII(wt) and DN-Cul1-5 proteins contain Flag-tag, which was used to detect the respective proteins by Western blotting. Asterisks (*) indicate degradation products of the DN-Cul proteins. Relative accumulation of the pVII(wt)Flag proteins is shown as the fluorescence signal ratio of pVII/Actin proteins. Hyphen (-) indicates non-Dox induced cells. (**B**) The pVII(K26R/K27R) and pVII(Δ24) protein levels are not changed by the DN-Cul3 protein overexpression. Expression of the pVII(wt)Flag, pVII(K26R/K27R)Flag, pVII(Δ24)Flag and DN-Cul3-Flag proteins were detected with Flag antibody in HEK293T cells. The ratio of the Flag-tagged pVII proteins to Actin signals (pVII/Actin) is shown after quantification of the fluorescence signals on the Western blot image. Lanes 6 and 7 are derived from the same Western blot image as lanes 1-5. Hyphen (-) indicates non-transfected cells. (**C**) DN-Cul3 blocks the pVII(wt)Flag protein decay. HeLa cells transiently expressing pVII(wt)Flag in the presence or absence of DN-Cul3-Flag (lanes 7-11) were treated with CHX for 3 hours (lanes 3 and 8), 6 hours (lanes 4 and 9), 9 hours (lanes 5 and 10) and 12 hours (lanes 6 and 11). The expressed proteins were detected with anti-Flag and anti-Actin antibodies. Relative accumulation of the pVII(wt)Flag protein is shown as the fluorescence signal ratio of pVII/Actin proteins. Hyphen (-) indicates non-transfected cells. (**D**) siRNA knockdown of Cul3 and Psmb1 in HEK293-pVII(wt)Flag cells results in an increase of the pVII(wt)Flag protein levels. After 36 hours of siRNA transfection the pVII(wt)Flag expression was induced with Dox for additional 14 hours. The expression of the proteins was detected by Western blotting with anti-Flag, anti-Cul3 and anti-Actin antibodies. Relative accumulation of the pVII(wt)Flag protein is shown as the fluorescence signal ratio of pVII/Actin proteins.

Our results so far indicated that the proteasomal protein degradation pathway might be involved in regulation of the pVII(wt) protein stability. To further dissect the mechanisms underlying the pVII(wt) protein stability, we were interested to find out the potential E3 ligases responsible in this process. For this purpose, we decided to evaluate the role of Cullin-RING E3 ubiquitin-ligases (CRL), as they have been considered as the most prominent class of ubiquitin-ligases [22]. Transient overexpression of the dominant-negative cullin (DN-Cul) proteins is an effective way to inactivate the function of endogenous cullins [32]. The DN-Cul proteins contain a C-terminal deletion, which eliminates their possibility to interact with the E2 ubiquitin-conjugating enzymes, thereby blocking the substrate protein ubiquitination and degradation. However, since the N-terminal domain is intact, the DN-Cul proteins are still able to bind to the substrate proteins [35]. Consequently, overexpression of DN-Cul1-5 in HEK293-pVII(wt)Flag stable cell line resulted in accumulation of the pVII(wt) protein, specifically in the presence of the DN-Cul1 and DN-Cul3 proteins (Figure 4A). As further evaluation revealed that the increase in the pVII(wt) protein signal was due to overlapping migration of the DN-Cul1 protein degradation product (Figure S2), we focused on the effect of DN-Cul3 on the pVII(wt) protein in the subsequent experiments. Importantly, DN-Cul3 stabilized the pVII(wt) protein (Figure 4B, lanes 2 and 3), whereas the pVII(K26R/K27R) and pVII(Δ24) proteins did not show similar accumulation pattern under the same experimental conditions (Figure 4B, lanes 4-7). Since the experiments presented so far did not address the direct involvement of Cul3 in pVII(wt) protein stability, we repeated the experiments with DN-Cul3 in the presence of cycloheximide in HeLa cells. Similarly to the previous experiments (Figures 4A and 4B), overexpression of the DN-Cul3 protein blocked the pVII protein decay after the *de novo* protein synthesis was inhibited with cycloheximide (Figure 4C, compare lanes 5 and 6 to lanes 10 and 11). To further confirm the involvement of Cul3 in the pVII protein stability, we reduced the endogenous Cul3 protein levels by siRNA treatment. Similarly to the DN-Cul3, Cul3 siRNA increased the pVII(wt) protein levels in HEK293-pVII(wt)Flag cell line (Figure 4D). The same cell line was also treated with siRNA against Psmb1, which is a catalytic subunit of the proteasome, to confirm the involvement of a functional proteasome in the control of the pVII(wt) protein stability (Figure 4D and Figure S3). Thus, our results pointed to the involvement of the Cul3-based CRL (CRL3) in the pVII(wt) protein stability control in HEK293T and HeLa cells. 

### Propeptide influences Cul3 association and nucleolar localization of the pVII(wt) protein

**Figure 5:**
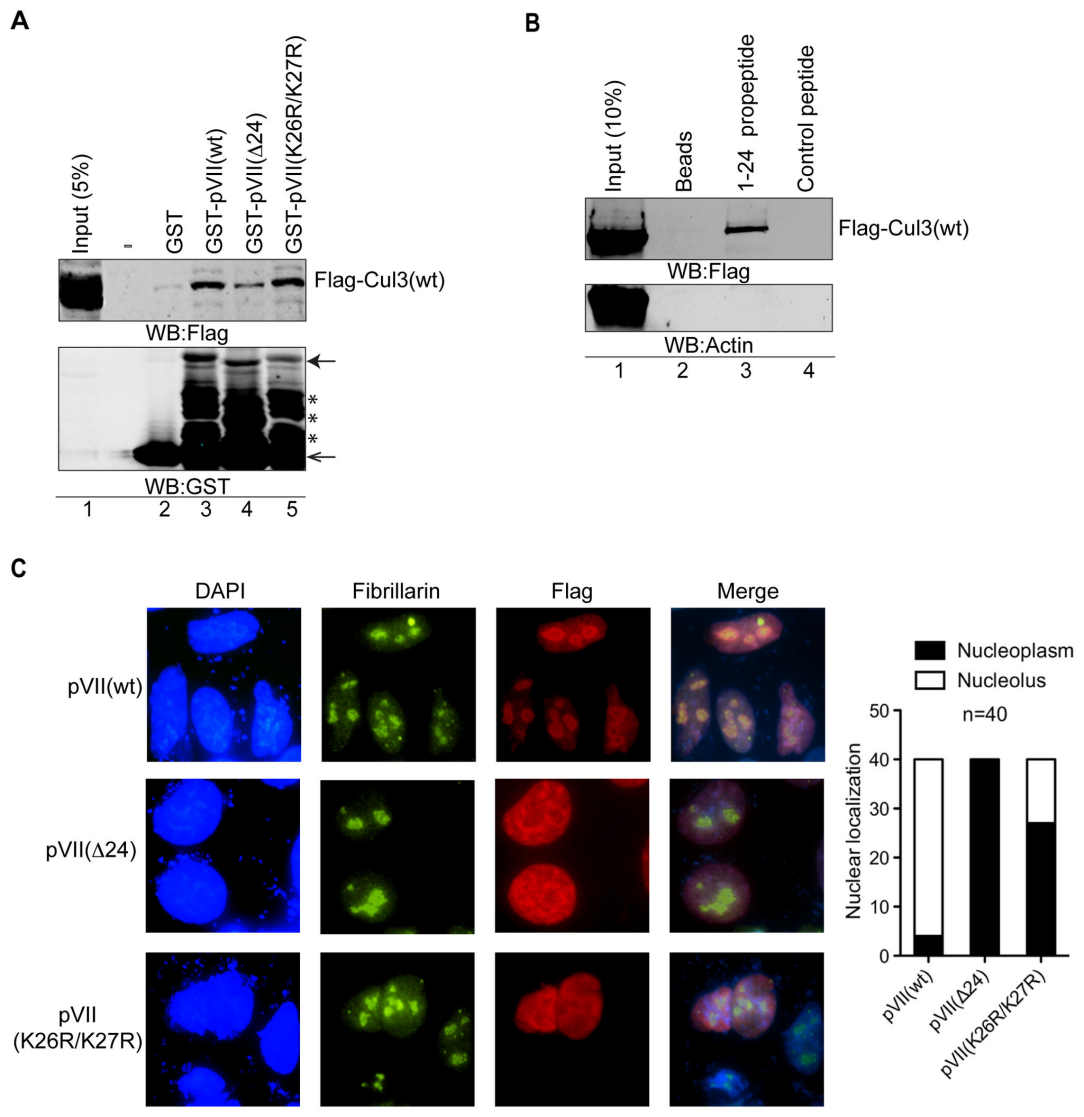
Propeptide influences Cul3(wt) association and nucleolar localization of the pVII(wt) protein. (**A**) The propeptide 1-24 sequence enhances Flag-Cul3(wt) interaction with the pVII protein. GST, GST-pVII(wt), GST-pVII(Δ24) and GST-pVII(K26R/K27R) were used in a GST-pull down assay with HEK293T whole cell lysate expressing Flag-Cul3(wt) protein. Filled arrow indicates the migration of the full-length pVII(wt), pVII(Δ24) and pVII(K26R/K27R) proteins. Non-filled arrow indicates migration of the GST protein. Asterisks (*) indicate the presence of truncated versions of the GST-pVII proteins. Hyphen (-) indicates an empty lane on the SDS-PAGE. (**B**) 1-24 propeptide interacts with the Flag-Cul3(wt) protein. Peptide pull-down assay with the synthetic 1-24 propeptide (lane 3) and control peptide (lane 4) was performed with HEK293T cell lysates expressing Flag-Cul3(wt) protein. Non-peptide coupled magnetic beads were also used as the specificity control (lane 2). Input represents 10% of the HEK293T cell lysates expressing Flag-Cul3(wt) protein. (**C**) Localization of the pVII(wt)Flag, pVII(Δ24)Flag and pVII(K26R/K27R)Flag proteins in the nucleus. HeLa cells were transfected with the respective plasmids for 24 hours and were subjected to indirect immunofluorescence analysis. The Flag-tagged pVII proteins and fibrillarin were simultaneously stained and visualized. Nuclei were counterstained with DAPI. Quantification of Flag-tagged pVII protein localization in HeLa cells (n=40) is shown on the right side of the Figure 5C.

The cullins function as the scaffold proteins by positioning the substrate proteins in close proximity to the E2 enzymes, which in turn couple the ubiquitin to substrate proteins [22]. Since our data indicated that Cul3 was involved in the control of pVII(wt) stability, we hypothesized that Cul3 can recognize pVII as the substrate protein. To test this hypothesis we conducted GST-pVII pull-down experiments with HEK293T cell lysates expressing the Flag-epitope tagged Cul3(wt) protein. As shown in Figure 5A, the GST-pVII and Flag-Cul3(wt) proteins interacted specifically (lanes 1 and 2). The interaction between pVII(wt) and Cul3(wt) was partially dependent on the propeptide sequence, as the pVII(Δ24) protein showed reduced interaction with the Flag-Cul3(wt) protein when compared to the pVII(wt) protein (Figure 5A, lanes 3 and 4). Interestingly, the pVII(K26R/K27R) protein showed a very similar affinity towards the Flag-Cul3(wt) as did the pVII(wt) protein (Figure 5A, compare lanes 3 and 5). To further show that the 24 amino acid propeptide sequence mediates the interaction with the Cul3(wt) protein, we performed a peptide pull-down experiment. For this purpose, we incubated magnetic beads-bound synthetic peptides with HEK293T cell lysate expressing the Flag-Cul3(wt) protein and monitored the Flag-Cul3(wt) binding capacity by Western blotting. Indeed, the 1-24 propeptide showed interaction with the Flag-Cul3(wt) protein, but not with the control actin protein (Figure 5B, lane 3). The interaction with the 1-24 propeptide was specific as a randomly picked control peptide and the magnetic beads did not show a detectable Flag-Cul3(wt) interaction (Figure 5B, lanes 2 and 4). Previous studies have established that pVII is a strictly nuclear protein [12,40,41]. Since our data pointed to the involvement of Cul3 in pVII(wt) protein stability, it became of interest to monitor the nuclear localization pattern of pVII(Δ24) and pVII(K26R/K27R), as these proteins showed an elevated protein stability compared to the pVII(wt) protein (Figure 3). To determine the pVII protein subcellular localization pattern we performed immunofluorescence experiments in pVII plasmid transfected HeLa cells. The results showed that all three proteins, pVII(wt), pVII(Δ24) and pVII(K26R/K27R) localized strictly to the nucleus as shown by the Flag antibody and DAPI stainings (Figure 5C). Interestingly, the pVII(wt) protein showed a distinct localization in the proximity of the nucleoli, defined by the cell staining with the nucleolus specific protein fibrillarin. In contrast, pVII(Δ24) and most of the pVII(K26R/K27R) expressing cells did not show a notable localization in the proximity of nucleoli (Figure 5C). A simple quantification further supported the nucleoplasmic localization pattern of the pVII(Δ24) and pVII(K26R/K27R) proteins (Figure 5C, right panel). 

### Cul3 is required for efficient adenovirus infection

**Figure 6:**
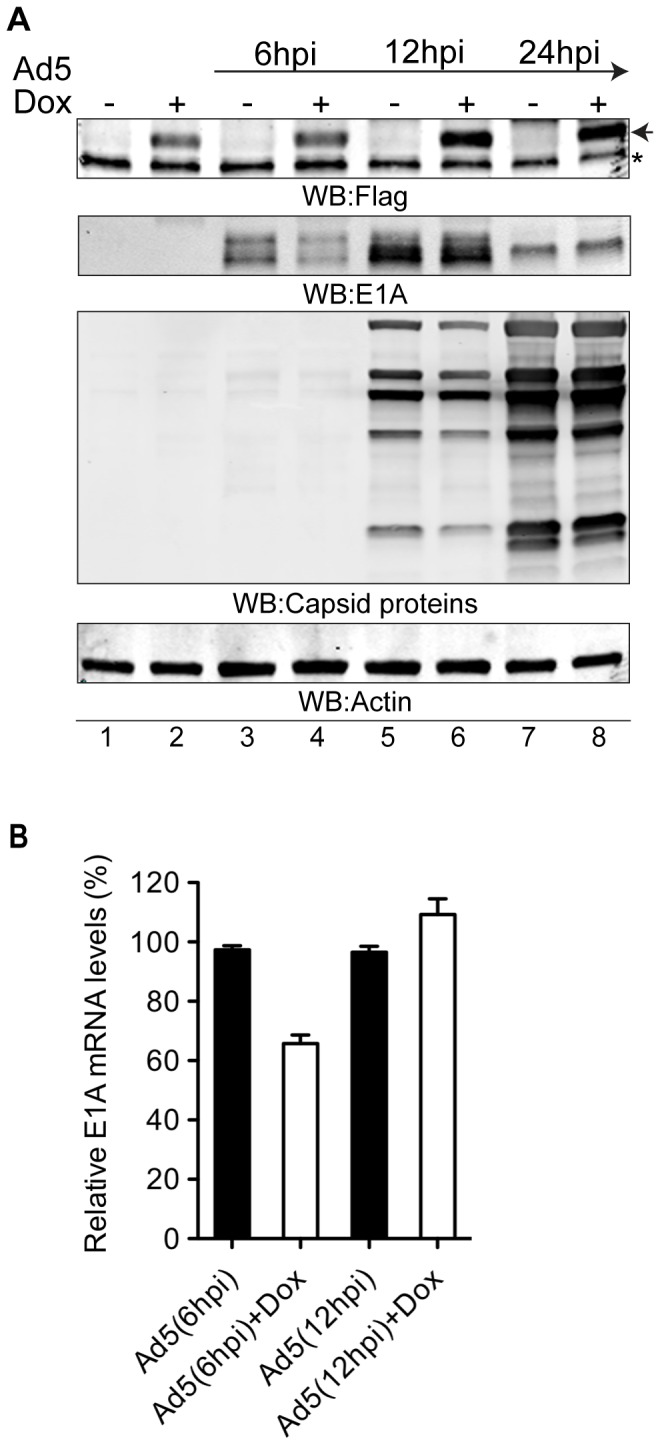
Cul3 controls adenovirus early E1A gene expression in infected cells. (**A**) DN-Cul3 reduces expression of the Ad5 E1A protein. Expression of the DN-Cul3-Flag protein was induced with Dox in HeLa-DN-Cul3-Flag stable cell line for 24 hours followed by Ad5 infection (MOI=5). Total cell lysates were prepared 6 hpi, 12 hpi and 24 hpi and the expression levels of the viral E1A, viral capsid proteins, Flag-tagged DN-Cul3 and Actin proteins were detected by Western blotting. Black arrow indicates the migration of the DN-Cul3-Flag protein, asterisk (*) indicates a non-specific protein detected with anti-Flag antibody (Sigma, F7425). Representative image from three independent experiments is shown. (**B**) DN-Cul3 reduces Ad5 E1A mRNA accumulation. E1A mRNA expression was detected by qRT-PCR from the HeLa-DN-Cul3-Flag stable cell lines undergoing the same treatments as in (A). Data is shown as the average from two independent experiments. E1A expression levels were normalized to the 18S rRNA levels. Hour post infection (hpi).

Based on our presented results it became of interests to evaluate the role of the Cul3 protein during the adenovirus infection. Therefore we generated HeLa stable cell line expressing the DN-Cul3-Flag protein as the response to the doxycycline-treatment. This cell line was used to monitor the accumulation of viral proteins after infection with Ad5 virus at different time points. As shown in Figure 6, the induction of the DN-Cul3-Flag protein with doxocycline reduced early viral E1A protein expression at 6 hpi (hours post-infection) (lanes 3 and 4). Similarly, the accumulation of some of the late viral structural proteins was slightly reduced in the presence of DN-Cul3-Flag after 12hpi (Figure 6A, lanes 5 and 6). The latter phenotype is probably due to reduced levels of the E1A protein, which is needed for the full-activity of other adenovirus transcription units [42]. To evaluate if the E1A protein decline at early time point of infection was due to the reduced early viral gene transcription, we analysed E1A mRNA levels in the same DN-Cul3-Flag overexpressing cell line. Indeed, the results from qRT-PCR showed the reduction of E1A mRNA levels after 6 hpi (Figure 6B), thereby correlating with the E1A proteins levels at the same time point (Figure 6A, lane 4). Similar reduction of the E1A protein and mRNA levels was not detectable after 12 hpi, indicating that low levels of the E1A at 6 hpi were able to revert the DN-Cul3-Flag effect during the late phase of infection. Taken together, our data indicated that the Cul3 protein might function as a positive factor for adenovirus early E1A gene expression in HeLa cells. 

## Discussion

Small DNA tumor viruses (adenoviruses, papillomaviruses and polyomaviruses) commonly target the ubiquitin-proteasome system to achieve optimal virus replication [43,44]. Extensive studies on human adenoviruses have shown that the viral E1B-55K/E4orf6 complex interacts with the Cul2- and Cul5-based E3 ubiquitin ligase (CRL2 and CRL5, respectively) complexes [45]. This mutual interaction results in the proteolytic degradation of several cellular proteins, such as p53, Mre11, DNA ligase IV, integrin α3, Tip60 and ATRX [24-29]. Furthermore, the Ad12 E4orf6 or Ad5 E1B-55K proteins can induce degradation of the cellular proteins TOPBP1 and Daxx without forming the complex with the respective Ad12 E1B-55K and Ad5 E4orf6 proteins [46,47]. 

In the present report, we provide experimental evidence that the Ad5 pVII(wt) protein can be considered as another adenovirus protein interfering with the CRL complexes via the scaffold Cul3 protein. In contrast to the E1B-55K and E4orf6 proteins, which to the best of our knowledge do not undergo proteolytic degradation by the CRL2 and CRL5 complexes, the pVII(wt) protein is proteolytically targeted by the CRL3 complex. Stabilization of the pVII(wt) protein in the presence of DN-Cul3 and specific interaction between the pVII(wt) and Cul3 proteins provide a proof for the potential interplay between these two proteins. Furthermore, our data indicate that the Ad5 propeptide sequence has a destabilizing effect on the pVII(wt) protein. Thus, the instability of the 1-24Gal4 fusion protein, faster decay rate of the pVII(wt) protein in the presence of cycloheximide and increased steady-state accumulation of the pVII(wt) protein after MG132 treatment are pointing to the instability of the proteins containing the propeptide sequence. In line with these observations we show that the propeptide sequence alone can contribute to the interaction with the Cul3 protein. All together, the presented data indicate that the propeptide has adopted the sequence constraints, which facilitate recruitment of the Cul3 protein. Closer inspection of the conserved Ad5 propeptide amino acid sequence showed that this 24 amino acid sequence is relatively rich in serines. The Cul3 protein functions as the scaffold protein in the CRL3 complex to position the substrate proteins in close proximity to the E2 ubiquitin–conjugating enzymes. To recognize the substrate proteins, Cul3 employs the so-called BTB-domain proteins as substrate-specific adaptors. The human genome encodes about 200 BTB domain proteins, which could be theoretically engaged in functional CRL3 complexes [48]. Interestingly, one of the best-characterized BTB domain proteins, named as the SPOP (Speckle-type POZ protein), has been shown to have a high affinity towards serine-rich motifs in the substrate proteins [49]. Therefore, the involvement of the SPOP as the Cul3 adaptor protein in recognition of the Ad5 propeptide sequence has to be considered.

Importantly, the Ad5 pVII(wt) protein contains two components, the propeptide sequence and the lysine residues K26/K27, which contribute to the protein stability regulation. Based on our results, we propose that these components function in a co-dependent manner. This is based on two following observations. First, the lysine K26/K27 mutations in the pVII(Δ24) protein, which lacks the propeptide sequence, did not increase the protein stability. This was in opposite to the precursor pVII(wt) protein, where the same point mutations increased the protein stability. Second, the pVII(K26R/K27R) protein interacted with the Cul3 protein as efficiently as did the pVII(wt) protein. In contrast, the pVII(Δ24) protein showed reduced interaction with the Cul3 protein, when compared to the pVII(wt) and pVII(K26R/K27R) proteins. Therefore, we suggest that the propeptide sequence functions as the primary substrate recognition component for the CRL3 complexes and that the lysine residues K26/K27 serve as the potential target sites for CRL3-mediated ubiquitination and proteasomal degradation.

The alignment of human Ad5 pVII(wt) amino acid sequence to other human serotype sequences revealed that the K26 and K27 residues are highly conserved among human adenoviruses, with exception of Ad4 and Ad7, which lack lysines at positions 26 and 27. Therefore, these lysine residues could also contribute to the pVII(wt) stability in other human adenovirus serotypes. It should be noted that we had difficulties to detect the ubiquitinated pVII(wt) protein in mammalian cells. The reasons might be due to rapid deubiquitination of the pVII(wt) protein during the protein sample manipulations or due to the low CRL3 complex ubiquitination activity in the cells used in our studies. Therefore, at present we can not provide direct evidence that the Ad5 pVII(wt) is modified by CRL3-mediated ubiquitination at the lysine residues K26 and K27, which would target the protein for proteasomal degradation. However, our data still confirms the involvement of these residues in the Ad5 pVII(wt) protein stability control.

 The presented data also indicate the involvement of the functional Cul3 protein during productive adenovirus infection. Interestingly, we show that inactivation of the Cul3 protein by dominant-negative approach affected viral E1A mRNA and protein accumulation during early phase of infection. The data indicated that the active Cul3 protein is involved during the early stages of virus infection to support efficient E1A gene expression. Theoretically, the functional Cul3 protein might act directly to achieve an efficient E1A transcription during early phase of infection. In line with this hypothesis is a recent work by Schreiner et al., (2012), where the authors showed that the transcriptional repressor protein Daxx inhibits E1A gene transcription [31]. Intriguingly, the Daxx protein has been shown to be ubiquitinated and proteasomally degraded by the Cul3-based CRL complex [36]. It is possible that during early times of adenovirus infection the CRL3 complex is involved in the proteasomal degradation and/or ubiquitin-modification of the Daxx protein present on the viral E1A transcription unit. This kind of interplay between CRL3 and Daxx could theoretically relieve silencing of the E1A transcription unit by Daxx during the early phase of infection. We are also aware of the published roles of the VI and Nedd4 proteins in counteracting Daxx-mediated E1A repression [31,50]. However, it is possible that the CRL3, VI and Nedd4 proteins target different Daxx functions during early time of the infection. An alternative, more speculative hypothesis, would suggest that Cul3 might play a role in the transport and escape of adenovirus particles from the endosomes in infected cells. This would be in line with the recent reports, suggesting that the CRL3 complexes could be responsible for ubiquitination and proteasomal degradation of proteins involved in the efficient endosomal transport of influenza A and vaccinia viruses in infected cells [51,52]. It is hypothetically feasible that the Cul3 protein, which has been detected in the early and late endosomal membranes [51], stimulates also adenovirus trafficking in the endosomal pathway [53] to achieve efficient nuclear accumulation of the viral genomes. 

 In conclusion, we show that the stability of the Ad5 pVII(wt) protein is regulated by the Cul3 protein targeting the propeptide sequence present in the precursor pVII(wt) protein. We also provide the evidence that functionally active Cul3 contributes to the efficient viral E1A gene expression during Ad5 infection. 

## Supporting Information

Figure S1 **Decay of the pVII(wt)Flag pVII(Δ24)Flag proteins.** Representative image used for the pVII(wt)Flag and pVII(Δ24)Flag protein decay calculations shown in Figure 2B. CHX treatment for 1 hour (lanes 3 and 10), 2 hours (lanes 4 and 11), 3 hours (lanes 5 and 12), 6 hours (lanes 6 and13) and 9 hours (lanes 7 and 14). Letter “M” denotes non-drug treated cells.
(TIF)

Figure S2 **Overlapping migration of the pVII(wt)Flag protein with a degradation product originating from the DN-Cul1-Flag protein.** Total cell lysates from transiently transfected HEK293T cells overexpressing the pVII(wt)Flag (lane 1) and DN-Cul1-Flag (lane 2) proteins were analysed by Western blotting. After detection of the proteins with anti-Flag antibody, an overlapping migration pattern of the pVII(wt)Flag protein with a degradation product of the DN-Cul1-Flag protein was observed. Lanes 1 and 2 are derived from the same Western blot image. Asterisks (*) indicate degradation products of the DN-Cul1 protein, arrows indicate the presence of the pVII(wt)Flag and DN-Cul1-Flag proteins.
(TIF)

Figure S3 **Reduction of the Cul3 and Psmb1 mRNA levels by siRNA treatment.** Total RNA was extracted from HEK293-pVII(wt)Flag cells after 36 hours of siRNA transfection and additional Dox-treatment for 14 hours. QRT-PCR was performed on random primed cDNA by using specific primers against Cul3 and Psmb1 (Table S1). The efficiency of Cul3 and Psmb1 siRNAs was compared to scrambled siRNA (Scr). Data is shown from a single experiment performed in triplicate and the expression data was normalized to 18S rRNA levels.
(TIF)

Table S1 **Primer sequences used in qRT-PCR experiments.**
(DOCX)
