# Supplementary figures and images for: Adenovirus Precursor pVII Protein Stability Is Regulated By Its Propeptide Sequence
Source: PLoS One. 2013 Nov 15;8(11):e80617. doi: 10.1371/journal.pone.0080617 (PMC3829898; doi:10.1371/journal.pone.0080617)

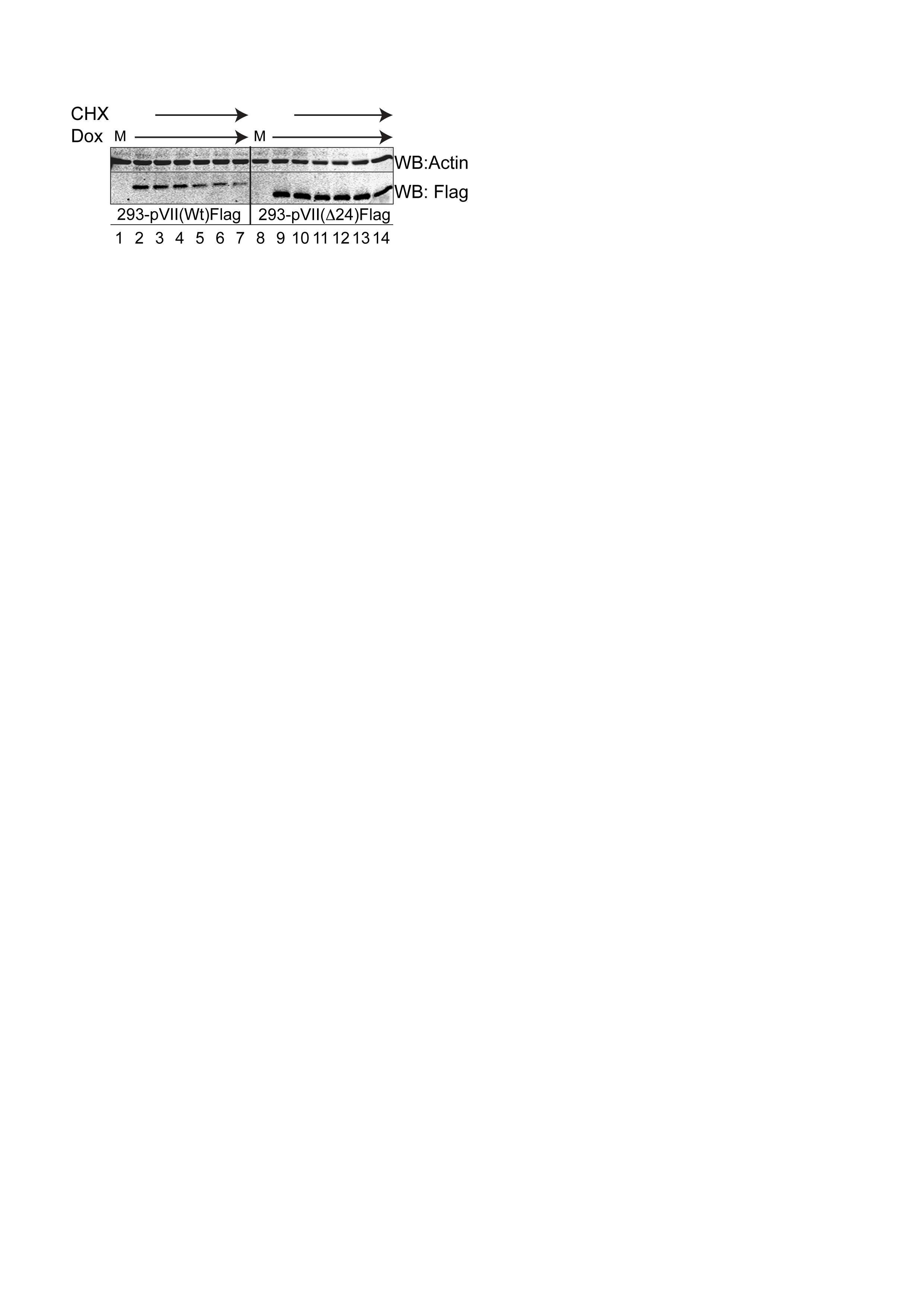

Supplement: Figure S1 — Decay of the pVII(wt)Flag pVII(Δ24)Flag proteins. Representative image used for the pVII(wt)Flag and pVII(Δ24)Flag protein decay calculations shown in Figure 2B. CHX treatment for 1 hour (lanes 3 and 10), 2 hours (lanes 4 and 11), 3 hours (lanes 5 and 12), 6 hours (lanes 6 and13) and 9 hours (lanes 7 and 14). Letter “M” denotes non-drug treated cells. (TIF) [file pone.0080617.s001.tif]

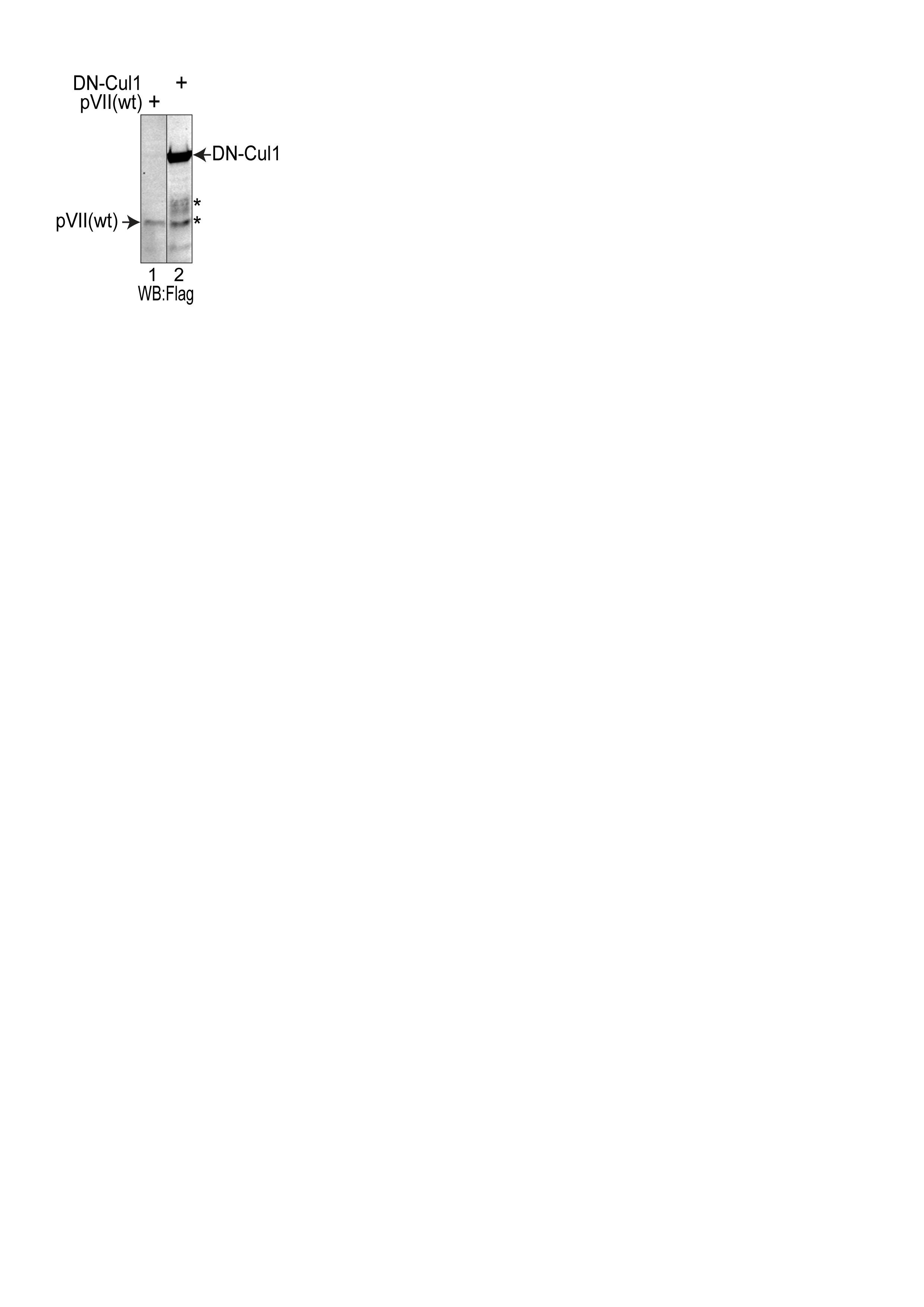

Supplement: Figure S2 — Overlapping migration of the pVII(wt)Flag protein with a degradation product originating from the DN-Cul1-Flag protein. Total cell lysates from transiently transfected HEK293T cells overexpressing the pVII(wt)Flag (lane 1) and DN-Cul1-Flag (lane 2) proteins were analysed by Western blotting. After detection of the proteins with anti-Flag antibody, an overlapping migration pattern of the pVII(wt)Flag protein with a degradation product of the DN-Cul1-Flag protein was observed. Lanes 1 and 2 are derived from the same Western blot image. Asterisks (*) indicate degradation products of the DN-Cul1 protein, arrows indicate the presence of the pVII(wt)Flag and DN-Cul1-Flag proteins. (TIF) [file pone.0080617.s002.tif]

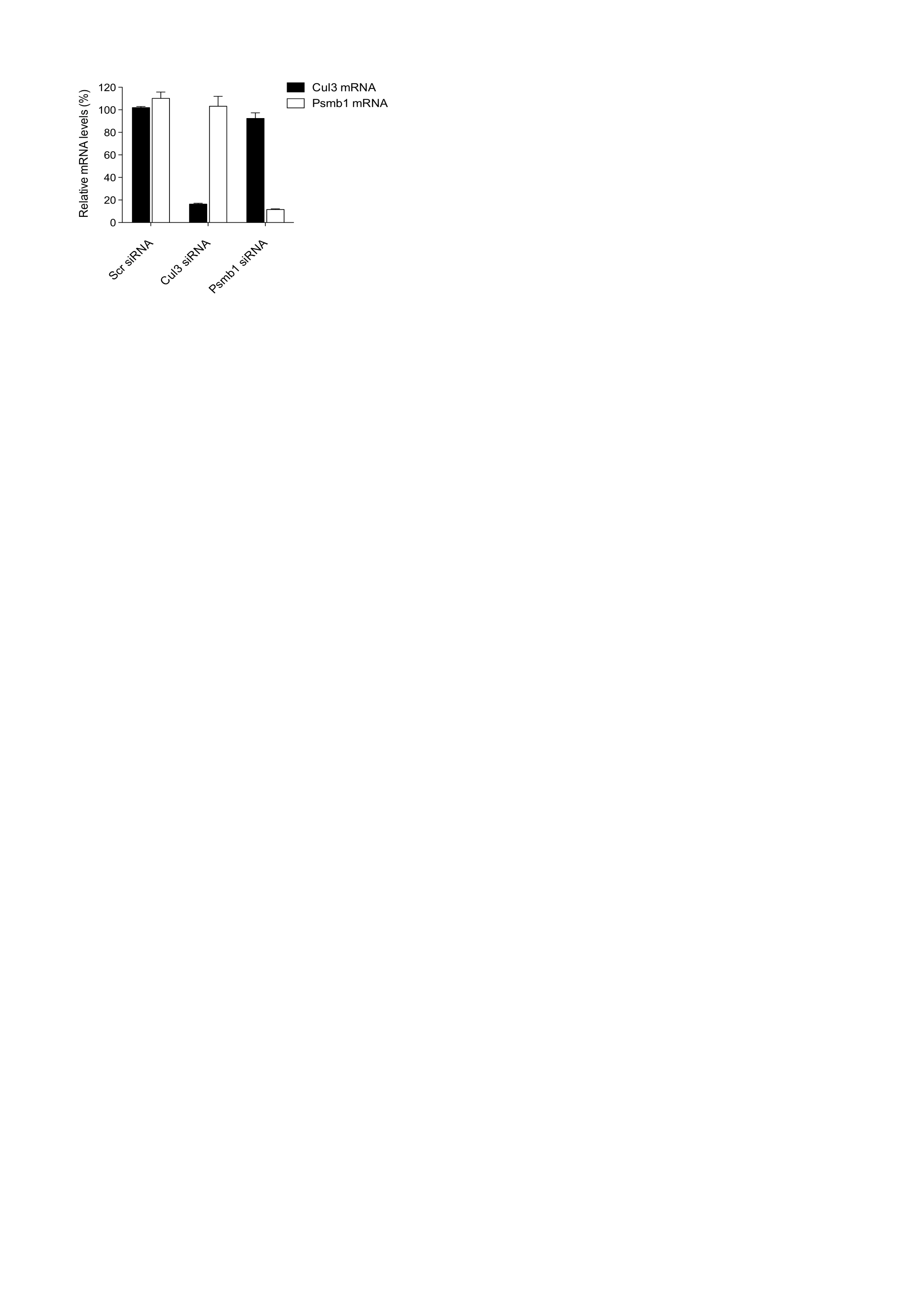

Supplement: Figure S3 — Reduction of the Cul3 and Psmb1 mRNA levels by siRNA treatment. Total RNA was extracted from HEK293-pVII(wt)Flag cells after 36 hours of siRNA transfection and additional Dox-treatment for 14 hours. QRT-PCR was performed on random primed cDNA by using specific primers against Cul3 and Psmb1 (Table S1). The efficiency of Cul3 and Psmb1 siRNAs was compared to scrambled siRNA (Scr). Data is shown from a single experiment performed in triplicate and the expression data was normalized to 18S rRNA levels. (TIF) [file pone.0080617.s003.tif]
