# Supplementary material for: Adenovirus Precursor pVII Protein Stability Is Regulated By Its Propeptide Sequence
Source: PLoS One. 2013 Nov 15;8(11):e80617. doi: 10.1371/journal.pone.0080617 (PMC3829898; doi:10.1371/journal.pone.0080617)
Supplement: Table S1 — Primer sequences used in qRT-PCR experiments. (DOCX) [file pone.0080617.s004.docx]

**Tabel S1. Sequences of the primers used in qRT-PCR experiments.**

18S rRNA forward 5´-TCGTCTTCGAACCTCCGACT-3´

18S rRNA reverse 5´-CCCCTCGATGCTCTTAGCTG-3´

Psmb1 forward 5´-CCTCCAGCCTTGAAGGAGTC-3´

Psmb1 reverse 5´-CAAGGCGCTTCTTTCCATAC-3´

Cul3 forward 5´- CCATGTCGAATCTGAGCAAA-3

Cul3 reverse 5´-TGCATTTTTCAGAAGGTCCC-3´

E1A forward 5'-CTTGGGTCCGGTTTCTATGC-3

E1A reverse 5'-CCCGTATTCCTCCGGTGATA-3´
